# Neurobehavioral functions and sleep architecture during polyphasic and monophasic short sleep schedules

**DOI:** 10.1093/sleep/zsag031

**Published:** 2026-02-05

**Authors:** Tiffany B Koa, June C Lo

**Affiliations:** Centre for Sleep and Cognition, Yong Loo Lin School of Medicine, National University of Singapore, Singapore; Human Potential Translational Research Programme, Yong Loo Lin School of Medicine, National University of Singapore, Singapore; Centre for Sleep and Cognition, Yong Loo Lin School of Medicine, National University of Singapore, Singapore; Human Potential Translational Research Programme, Yong Loo Lin School of Medicine, National University of Singapore, Singapore; Department of Medicine, Yong Loo Lin School of Medicine, National University of Singapore, Singapore

**Keywords:** cognitive performance, mood, partial sleep deprivation, polyphasic sleep, sleepiness

## Abstract

**Study Objectives:**

To investigate how sleep architecture and neurobehavioral functions change during a polyphasic short sleep schedule and to compare these responses to those of a monophasic short sleep schedule with the same total sleep opportunity, as well as to those of a well-rested control group.

**Methods:**

Forty healthy young adults (18 males, age: 18-35) were assigned to either the monophasic short sleep group, which had a single 2-h sleep opportunity, or the polyphasic short sleep group, which followed the “Uberman” sleep schedule and had six 20-min sleep opportunities distributed evenly across 24 h (1 every 4 h). Polysomnography was conducted during every sleep opportunity. Neurobehavioral functions were assessed at baseline (before the sleep opportunity manipulation started) and 6 times thereafter (once every 4 h).

**Results:**

Both short sleep groups experienced greater subjective sleepiness, poorer vigilance, and lower positive mood as compared to a well-rested control group. Relative to the monophasic short sleep group, the polyphasic short sleep group showed greater vigilance impairment, particularly in the morning. This was accompanied by greater reductions in total sleep time, longer total sleep onset latency and wake after sleep onset, as well as greater proportions of N1 and N2 but lower proportions of N3 sleep in the polyphasic short sleep group relative to the monophasic short sleep group.

**Conclusions:**

In young adults, the “Uberman” polyphasic sleep schedule substantially reduces total sleep duration and sleep efficiency, even when compared to a monophasic sleep schedule with the same overall sleep opportunity, and may be associated with poorer neurobehavioral performance.

**Statement of Significance**

Polyphasic short sleep has been proposed to help retain a “scientifically required amount” of SWS and REM sleep, and optimize performance and mood despite the extended wake duration. We found that not only did the “Uberman” sleep schedule drastically shorten SWS and REM durations over a 24-h period, and degrade alertness, vigilance, and mood during the daytime, but its associated neurobehavioral deficits during the biological night, when one is normally asleep, were also prominent. Therefore, individuals will likely struggle cognitively and emotionally during both day and night, at least when they start adopting this sleep schedule. The long-term impacts of polyphasic sleep on sleep, daily functioning, and health remain to be extensively characterized.

## Graphical Abstract

**Graphical Abstract:**
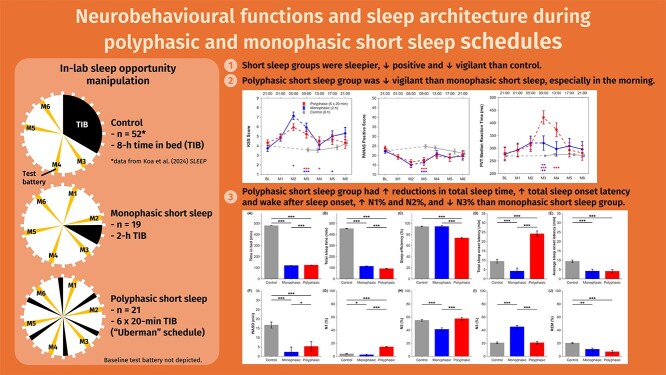

## Introduction

Short sleep is common in many industrialized societies, with 26–74% of adults sleeping less than the minimum age-specific recommended duration of 7 h [1–3]. Many students and working adults struggle to sleep the recommended amount [4, 5] because of competing wake activities [6, 7]. In fact, some actively search for short sleep schedules to maximize their wake duration in order to cope with demands from their study and work. Polyphasic sleep—deliberately distributing multiple sleep episodes over a 24-h day, typically with the goal of limiting total time-in-bed (TIB) to maximize wake duration [8]—has been claimed by its advocates to enable optimized performance, and improved productivity, memory, and mood despite the short sleep durations [9]. Various polyphasic sleep schedules have been proposed, ranging from a longer core sleep episode at night combined with one or multiple short naps to only naps of similar durations [9]. Although the prevalence of polyphasic sleep has not been extensively investigated, a study has shown that having multiple sleep bouts is common in a culture where each day, individuals have multiple obligatory prayers, including one before sunrise: 28% of the sample slept at least 3 times a day, 35% supplemented their nocturnal sleep with a daytime nap, and 11% had 2 sleep episodes at night [10]. Anecdotal reports also show that attempts to adopt polyphasic short sleep schedules are common [8].

Neurobehavioral functions during polyphasic sleep have, in a way, been characterized in a few studies that involved comparisons between elevated homeostatic sleep pressure induced by a night of total sleep deprivation and lower sleep pressure by introducing multiple nap episodes. These studies, in general, found that the nap opportunities helped reduce sleep loss-induced neurobehavioral deficits [11, 12]. For example, relative to total sleep deprivation, a 40-h polyphasic sleep schedule with adequate opportunity for sleep—10 cycles of 80-min sleep and 160-min wake (sleep:wake ratio = 1:2)—helped maintain alertness and sustained attention, while reducing thalamic and other subcortical responses [12]. In these studies, the sleep:wake ratio used enabled the individuals to have a total of 8-h TIB per 24 h, which is within the recommended sleep duration range [4], and hence, is different from the typical goal of polyphasic sleep proponents and practitioners to shorten sleep duration. Another limitation of these studies is that outcomes in the polyphasic sleep (or multi-nap) condition were not compared to a monophasic sleep schedule with the same total TIB per 24 h.

Several studies have addressed the latter limitation, although the total sleep opportunities over a 24-h period were still at least 8 h. For example, in a dated study on 1 participant who had a nightly 8-h TIB for the first month and then had a 3-h TIB after each 3-h wake period in the second month, negligible differences were found in multiple cognitive and motor functions between the 2 months [13]. In contrast, a study involving recurrent cycles of 60-min wake and 30-min sleep showed increased depression scores on the first day, suggesting that splitting sleep up into multiple short bouts per se, rather than sleep deficiency, may have a negative impact on mood [14]. Adopting this kind of polyphasic sleep schedule for 5.5 or 6 days resulted in frequent sleep onset rapid eye movement (REM) periods, and a 24-h fluctuation of subjective sleepiness, which peaked around early morning and the first half of morning [15]. Similar circadian patterns were found for subjective sleepiness, vigilance, and mood over 50 to 55 h of 60-min sleep and 120-min wake, and importantly, mood deteriorated on the second day, highlighting again the influence of polyphasic sleep on emotional regulation [16]. Circadian modulation of REM sleep was prominent in a 10-day study involving recurrent cycles of 1-h sleep and 2-h wake [17]. When the impact of polyphasic sleep was compared to a well-rested baseline of 8-h TIB, cycles of 60-min sleep and 160-min wake led to lower proportions of Stage 2 and REM sleep, no change in the proportion of Stage 3 sleep, and higher proportions of Stage 1 and Stage 4 sleep and importantly, wake, thereby, reducing sleep efficiency substantially from 94.2% to 61.0% [18]. In a forced desynchrony protocol with a sleep:wake ratio of 1:2, where participants had either one 9.33-h TIB, or 2 cycles of 4.67-h TIB and 9.33-h wake, every 28 h, the 2 sleep episodes in the biphasic sleep schedule resulted in longer total sleep time (TST), shorter wake after sleep onset (WASO), and more slow wave sleep (SWS), although sleep latency and Stage 1 duration increased relative to the monophasic sleep schedule [19]. However, others have reported that total durations of SWS and REM sleep were shorter during eight 1-h nap episodes across 24 h relative to a nightly 8-h TIB [17]. Therefore, when total sleep opportunities over a 24-h period likely will enable individuals to have sufficient sleep, polyphasic sleep will lead to poorest subjective sleepiness, vigilance, and mood in the early morning hours, with mood being the most vulnerable neurobehavioral domain; furthermore, proportion of N1 may increase, and REM may decrease, while effects on SWS and WASO are not consistent.

As for studies where the polyphasic sleep schedule did not provide sufficient total sleep opportunities, comparisons against a group that had the age-appropriate sleep opportunity at night will portray how polyphasic sleep altered neurobehavioral functions and sleep, while comparisons against a monophasic short sleep group of the same total TIB per 24 h will address whether the neurobehavioral and sleep changes during polyphasic short sleep could be attributed to splitting sleep up into multiple episodes. In a 15-day study on adolescents, we provided head-to-head comparisons of neurobehavioral responses to a monophasic (6.5-h nocturnal TIB) and a biphasic (5-h nocturnal TIB plus 1.5-h afternoon nap) short sleep schedule, both of which had the same total TIB across 24 h [20], against a group that received the age-appropriate sleep opportunity of 9 h every night. We found optimal and stable neurobehavioral functions in the well-rested control group, and importantly, less neurobehavioral deficits in the biphasic than the monophasic short sleep group. Polysomnographic data were also collected [20]: relative to monophasic sleep, biphasic sleep slightly shortened total durations across 24 h of TST, N2, and REM sleep. Total N3 duration was similar under both schedules, but biphasic sleep lengthened nocturnal N2 onset latency and reduced SWA, suggesting that having multiple sleep bouts across the day lowered homeostatic sleep pressure. This may explain why this sleep schedule induced less neurobehavioral impairment than monophasic short sleep. Although some might not recognize this biphasic sleep schedule as a variation of polyphasic sleep, because the daytime nap opportunity could be treated as supplementing the short nocturnal sleep episode [8], the comparisons against the monophasic short sleep group with the same total TIB over a 24-h period shed light on the effects of splitting up a nocturnal sleep opportunity which is below the recommended duration on sleep and neurobehavioral responses.

As mentioned, polyphasic short sleep schedules can go beyond a biphasic one and involve multiple naps of the same duration distributed across the 24-h day [9]. Polyphasic sleep advocates have claimed that following the proposed timing and duration of each sleep/nap episode helps retain a “scientifically required amount” of SWS and REM sleep, and reduce some “non-essential sleep” despite sleep professionals never having specified the amounts of SWS and REM sleep needed, and research having shown that N2 sleep is not “non-essential” as its characteristic feature—sleep spindle—is implicated in intelligence and memory consolidation [21, 22]. Little is actually known regarding sleep responses during these polyphasic sleep schedules. In Rosenblum et al.’s recent study which was initiated by participants with high intrinsic motivation and commitment to follow the “Uberman” sleep schedule (i.e. six 20-min sleep opportunities per 24 h) for 8 weeks, all but 1 participant withdrew within the first month, and polysomnographic sleep data during polyphasic sleep were collected from the only participant who managed to follow the schedule for 5 weeks [23]. Relative to this individual’s baseline 8-h sleep opportunity, the proportion of REM sleep was found to be reduced by 7%, while the proportion of SWS increased by 4% at the end of the 5-week polyphasic sleep period; limited changes were found for N1 and N2 proportions [23].

Other than sleep responses, neurobehavioral responses to polyphasic short sleep are also not well characterized. In Rosenblum et al.’s study, neurobehavioral assessments at baseline and after polyphasic sleep were conducted on the participant who followed the “Uberman” schedule for 5 weeks [23]. This individual’s vigilance deteriorated to the same extent as the 8-h nocturnal TIB control group, and the changes in other neurobehavioral functions—declarative memory, procedural memory, fluid reasoning, and manic/hypomanic and depressive symptoms—were also negligible [23]. On the other hand, in an earlier study [24], the polyphasic sleep group that had three 80-min naps distributed across 24 h (and the 8-h group) used a less conservative decision criterion in a vigilance task than the 4-h monophasic sleep group. An observational study has also reported that polyphasic sleep was associated with higher levels of daytime sleepiness [10]. Interestingly, in solo and double-handed ocean sailing races where most sailors had polyphasic sleep with each sleep episode lasting 20–120 min and overall TST of 6.3 h, short sleep was associated with better race performance; thus, polyphasic sleep, or perhaps the longer wake durations involved, enabled these sailors to finish the race more quickly [25].

To better understand the impact of polyphasic short sleep on sleep architecture and neurobehavioral functions, in this study, we investigated how sleep, cognitive functions, sleepiness, and mood changed during the “Uberman” sleep schedule, and these responses were compared with a monophasic short sleep group that had the same total TIB of 2 h per 24 h, as well as a control group with an 8-h TIB from a previous study [26].

## Materials and Methods

### Participants

Forty-five participants were recruited through the National University of Singapore’s online research recruitment platform, social-networking sites, and word of mouth. They completed an online screening questionnaire to ensure they met the following selection criteria: (1) were between 18 and 35 years of age, (2) had a body mass index (BMI) between 18.5 and 24.9 kg/m^2^, (3) did not suffer from sleep disorders, chronic physical illnesses or mental disorders (based on self-report, not categorized as having a high risk of sleep apnea using the Berlin Questionnaire [27], fell within the normal to mild range for all the subscales of the Depression, Anxiety and Stress Scale – 21 items [28], Epworth Sleepiness Scale (ESS) [29] score < 12, Insomnia Severity Index (ISI) [30] score < 8, and Pittsburgh Sleep Quality Index (PSQI) [31] global score < 6), (4) had a daily average TIB of at least 6 h according to their self-report and hence, were not habitual short sleepers, (5) did not have an extreme chronotype (Morningness-Eveningness Questionnaire [32] score: 31–69), (6) were not shift workers, (7) did not smoke, (8) did not typically consume more than 4 cups of caffeinated beverages per day, (9) did not typically consume more than 13 units of alcohol per week, and (10) did not travel across more than 2 time zones a month prior to the study.

Two participants withdrew prior to the start of the experiment due to personal reasons, and 2 participants in the polyphasic short sleep group and 1 participant in the monophasic short sleep group withdrew mid-study due to sleepiness. Therefore, 40 participants (mean age ± *SD*: 23.31 ± 3.37; 18 males) were included in the final analyses.

All participants received financial reimbursement for their participation. This study was approved by the Institutional Review Board of the National University of Singapore.

### Protocol

Three nights prior to the experiment, participants were instructed to adhere to an 8-h nocturnal sleep schedule that required them to wake up at their self-reported habitual wake time. This same wake time was used during the experiment itself. This was to facilitate circadian entrainment as well as to prevent any effects of prior sleep loss. To check for compliance, participants’ sleep was monitored using actigraphy and sleep diaries. During this period, participants were not permitted to take any naps or consume any alcoholic or caffeinated food and beverages.

**Figure 1:**
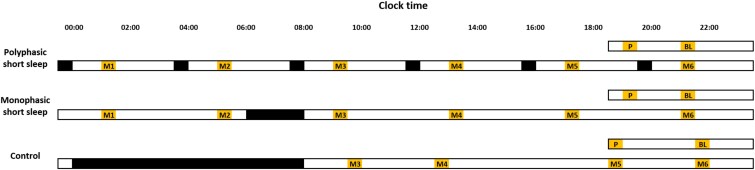
Experimental protocol. The raster plot depicts the study protocol. Participants were randomized into the polyphasic and the monophasic short sleep groups. Both groups arrived at the laboratory in the evening. Their sleep opportunities (black bars) were manipulated over a 24-h period: The polyphasic group had six 20-min sleep opportunities spaced 4 h apart, while the monophasic group had a single 2-h sleep opportunity. The third sleep opportunity of the polyphasic sleep group and the sleep opportunity of the monophasic sleep group ended at the participant’s habitual wake time. Sleep timings illustrated here are based on a habitual wake time of 08:00. A computerized test battery was administered 8 times for neurobehavioral assessment (yellow bars). The first one was for practice purposes (P), and these data were excluded from analyses. The remaining test batteries were administered every 4 h. The baseline (BL) test battery was conducted before the sleep opportunity manipulation started. Thereafter, each test battery during the manipulation period (M1–M6) was implemented 1 h after the polyphasic short sleep group’s scheduled wake time to minimize the impact of sleep inertia. The test battery timings were identical for the monophasic short sleep group. Test battery data collected at similar times of day on the first and second days of a 16-day protocol [26], when the scheduled sleep period was 8 h at night and ended at the participants’ habitual wake time, were extracted to benchmark neurobehavioral functions in a well-rested state.

The experiment was conducted at the Centre for Sleep and Cognition, National University of Singapore. Participants arrived at the sleep laboratory in the evening, approximately 11 h after their habitual wake time. Upon arrival, they were informed of the group they were randomly assigned to. Participants stayed in the sleep laboratory for the next 29 h during which the monophasic short sleep group (*n* = 19) was given a 2-h sleep opportunity that ended at their habitual wake time, while the polyphasic short sleep group (*n* = 21) had six 20-min sleep opportunities, one every 4 h, with the first one starting at 8 h 20 min before their habitual wake time and the third sleep opportunity ending at the participant’s habitual wake time (Figure 1). Polysomnography (PSG) was conducted for all sleep episodes.

During the experiment, a battery of cognitive tests and psychological scales was administered 8 times. The first (practice) test battery was to familiarize the participants with all the tests again, since they had already encountered this test battery during their previous visit to the laboratory a few days ago. These data were not included in any analyses. The second test battery was administered 11 h prior to the participants’ habitual wake time and was used as the baseline (BL). Thereafter, the remaining 6 test batteries were implemented every 4 h during the period of TIB manipulation (M1–M6). Note that these test batteries were administered 1 h upon awakening from each of the 20-min sleep periods of the polyphasic short sleep group, and for the monophasic short sleep group, the M3 test battery was administered 1 h after these participants woke up from their 2-h sleep opportunity (Figure 1). Test battery data from our previous study [26] were used to illustrate the levels of subjective alertness, cognitive performance, and mood achieved at a well-rested state. More specifically, this additional group of 52 participants met similar selection criteria and took part in a 16-day stay-in protocol in our laboratory. In the first night, all of them had an 8-h TIB that ended at their habitual wake time. We extracted their test battery data from the first evening and the following day at timings similar to the current experiment: 30-min difference for all the test batteries, except for M5, when the difference was 1.5 h (Figure 1).

In the sleep laboratory, each participant was assigned a sound-attenuated sleep room without windows, where they slept and completed all the test batteries. Outside of scheduled experimental tasks, meal times, and sleep periods, participants were largely permitted to spend their time as they wished (e.g. working, studying, watching shows, interacting with other participants/research staff). They were not allowed to sleep outside the scheduled period(s), perform strenuous physical activity, or leave the laboratory premises. During their waking periods, they were encouraged to stay in a common area that received natural and artificial light. Participants were monitored by the research staff at all times.

### Polysomnography

Electroencephalography (EEG) was performed using a SOMNOtouch recorder (SOMNOmedics GmbH, Randersacker, Germany) on 3 channels (C3, F3, and O1 in the International 10–20 system). The contralateral mastoid (A2) was used as reference. Electrodes placed at Cz and Fpz were used as common reference and ground electrodes, respectively. Electrooculography (EOG) and submental electromyography (EMG) were also used. Impedance was kept below 5 kΩ for EEG and 10 kΩ for EOG and EMG electrodes. Signal was sampled at 256 Hz and filtered between 0.2 and 35 Hz for EEG, and between 0.2 and 10 Hz for EOG. Sleep stages and artifactual epochs were scored every 30 s using an automatic PSG-scoring software [33] (Neurobit PSG, Neurobit Inc., NY, United States) in conjunction with the FASST toolbox [34] (http://www.montefiore.ulg.ac.be/~phillips/FASST.html) and visually checked by trained technicians, following criteria set by the American Academy of Sleep Medicine Manual for the Scoring of Sleep and Associated Events [35]. Variables of interest included TIB, TST, durations of each sleep stage, WASO, sleep onset latency (defined as the number of minutes taken to reach the first epoch of any sleep stage from lights off), and sleep efficiency. Furthermore, for the polyphasic sleep group, total TIB, TST, duration of each sleep stage, WASO, and sleep onset latency, together with the averages of sleep onset latency and sleep efficiency, across the 6 sleep episodes were derived. Percentage of TST spent in each sleep stage was derived for all groups.

### Computerized test battery

The computerized test battery had been used in multiple previous studies by our group to examine the impact of sleep deprivation on neurobehavioral functions [20, 26, 36–39] and consisted of 7 tasks that were administered in the following order: the Karolinska Sleepiness Scale (KSS) [40], the Symbol Digit Modalities Test (SDMT) [41], the verbal 1-back and 3-back tasks [42], the Mental Arithmetic Test (MAT) [43], the Positive and Negative Affect Schedule (PANAS) [44], and the Psychomotor Vigilance Task (PVT) [45]. These tasks were programmed in E-Prime 2.0 (Psychology Software Tools) and were conducted on identical laptops (Acer Aspire E11, Acer Inc, Taipei, Taiwan). Each test battery session lasted for approximately 25 min. Participants were administered the test batteries in their individual sleep rooms. During the test batteries, participants were monitored by the research staff via the closed-circuit television camera in their rooms, and they were required to wear earphones to minimize distractions and to allow beeping tones to be delivered in some of the tests in cases of non-response.

The KSS [40] was used to assess the participant’s current level of subjective sleepiness. Participants had to rate how alert they were feeling at that moment on a Likert scale that ranged from 1 (extremely alert) to 9 (very sleepy, great effort to keep awake, fighting sleep).

The SDMT [41] was used to measure speed of processing. In this 2-min task, participants were shown at the top of their computer screen a key consisting of 9 symbols, with each symbol paired with a corresponding number (1–9). For each trial, a target symbol was displayed below the key. Participants had to respond by pressing the corresponding number on the keyboard as quickly and as accurately as possible. If no response was detected within 15 s, the computer played a beeping tone until the participant responded. The number of correct responses was used as the outcome measure.

The verbal *n*-back tasks [42] were used to assess working memory and executive function. For both the 1-back and the 3-back tasks, alphabet letters were presented sequentially for 1 s with an inter-stimulus interval of 3 s. Participants had to determine whether the current stimulus displayed on-screen matched the stimulus that was presented one (1-back) or three (3-back) items ago. The match-to-mismatch ratio for the presented stimuli was 8:24. To quantify performance, a non-parametric measure of sensitivity (*A’*) was used [37]. *A’* indicated how well participants could discriminate between matches and mismatches, and ranges from 0 to 1, with 0.5 indicating chance-level performance. *B″_D_* is a measure of whether participants (a) tended to provide a “Yes” response and indicate the stimuli matched, i.e. they were liberal and more likely to detect matches when they were actually present (*B”_D_* < 0), (b) tended to provide a ‘No’ response and indicate the stimuli did not match, i.e. they were conservative and less likely to detect matches when they were actually present (*B”_D_* > 0), or (c) were neutral in their tendency to provide “Yes” and “No” responses (*B”_D_* = 0).

The MAT [43] was also used to measure speed of processing. Participants needed to accurately sum up as many pairs of 2-digit numbers as they could within 4 min. Similar to the SDMT, if participants did not respond within 15 s, they were presented with a beeping tone until they submitted a response. The number of correct responses was used as the outcome measure.

The PANAS [44] was used to assess the participant’s current levels of positive and negative mood. Participants were presented with 10 adjectives for positive mood and 10 adjectives for negative mood in random order. They had to rate each adjective on a Likert scale that ranged from 1 (very slight or not at all) to 5 (extremely), to indicate the extent to which each word described their feelings at that moment. The total scores for both the positive and negative affect subscales were derived.

A 10-min PVT [45] was used to assess vigilance. In this task, a counter was displayed in the center of the computer screen and started counting up at random intervals (varying between 2 and 10 s). Whenever the counter began, participants had to respond as quickly as possible by pressing the spacebar. If no response was detected within 10 s upon stimulus presentation, a short beeping tone was presented. The outcome of interest was median reaction time, mean speed (i.e. inverse of the mean reaction time), and the number of lapses (i.e. responses exceeding 500 ms) due to their sensitivity to sleep restriction [46].

### Statistical analyses

Statistical analyses were conducted using JASP (Version 0.19.3) and SAS Studio (SAS Institute Inc., Cary, NC, United States). To determine whether any of the screening parameters differed among the 3 groups, ANOVAs or chi-squared tests were performed. Where the homogeneity of variance assumption was not met, Welch’s ANOVA was used instead.

To verify the effectiveness of our sleep opportunity manipulation, we used ANOVA to ascertain the effect of group on TIB. Group differences in TST and the duration of each sleep stage were also tested. In these between-group analyses, for the polyphasic short sleep group, the sum of most of the sleep measures across the six 20-min sleep opportunities was used, except for sleep onset latency, which was indicated by both the total and the average across the 6 sleep opportunities, and sleep efficiency, which was indicated by the average across the sleep opportunities. Bonferroni correction was applied to all post hoc comparisons between groups. Where the homogeneity of variance assumption was not met, Welch’s ANOVA and Games-Howell post hoc tests were used instead. For the polyphasic short sleep group, to examine how the sleep measures changed across the sleep opportunities, general linear mixed models with PROC MIXED were conducted.

Regarding the neurobehavioral measures, since there were no data from the control group for sessions M1 and M2 given that these participants were having their scheduled sleep period, i.e. data were not “missing” at random, instead of using general linear mixed models on all 3 groups, 3 sets of analyses were conducted: (1) general linear mixed models with PROC MIXED examining the effects of group (monophasic short sleep group vs polyphasic short sleep group), session (from BL to M6), and group × session to investigate whether the 2 short sleep groups differed in neurobehavioral functions in each session, and whether changes across sessions were significant for each short sleep group, (2) general linear mixed models with PROC MIXED to determine whether neurobehavioral functions changed across sessions (BL to M3–M6) or remained stable for the control group, and (3) independent-samples *t* tests on the estimated means derived from the general linear mixed models to study whether neurobehavioral functions of each of the 2 short sleep groups differed from those of the control group at each available time point (BL and M3–M6). In all the general linear mixed models, contrasts of the estimated means were examined for within-group changes across sessions and between-group differences.

## Results

### Sample characteristics

The 3 groups did not differ in age, sex distribution, caffeinated drink and alcohol consumption, levels of stress, levels of daytime sleepiness, symptoms of insomnia, morningness-eveningness preference, self-reported habitual sleep durations and timings, and PSQI global score (all *p* > .06; Table 1). The group differences in BMI, alcohol consumption, and symptoms of anxiety were numerically small (*p* < .04).

**Table 1:** Demographics and habitual sleep characteristics for all groups

	***Monophasic***		***Polyphasic***		***Control***			
	**Mean**	**SE**	**Mean**	**SE**	**Mean**	**SE**	***F* /ꭓ** ^**2**^	***P***
*n*	19	-	21	-	52	-	-	-
Age (y)	23.09	0.72	23.50	0.79	22.75	0.23	0.65	.52
Sex (% males)	42.11	-	47.62	-	48.08	-	0.21	.90
BMI	22.62	0.45	21.84	0.46	20.97	0.28	5.03	**.009**
Caffeinated drinks per day (cups)	0.66	0.13	0.86	0.21	0.77	0.12	0.27	.76
Alcohol consumed per week (units)	1.39	0.55	0.16	0.12	0.74	0.21	4.72	**.01**
DASS-21								
Depression score	2.00	0.57	4.00	0.83	2.23	0.36	2.18	.13
Anxiety score	1.58	0.45	3.33	0.46	1.73	0.31	4.81	**.01**
Stress score	2.53	0.81	4.86	1.07	2.85	0.48	1.71	.20
ESS score	5.84	0.59	6.71	0.61	5.35	0.47	1.45	.24
ISI score	2.79	0.60	3.62	0.73	3.56	0.42	0.51	.60
MEQ score	46.95	1.37	48.10	2.19	46.87	0.94	0.13	.88
PSQI								
Average TIB (h)	8.08	0.16	8.04	0.17	8.00	0.10	0.08	.92
Average TST (h)	7.62	0.17	7.66	0.17	7.47	0.10	0.64	.53
Bedtime (clock time)	00:25	00:14	00:23	00:13	00:32	00:08	0.23	.80
Wake time (clock time)	08:30	00:13	08:25	00:16	08:32	00:09	0.08	.92
Global score	3.11	0.32	2.71	0.32	2.96	0.22	0.33	.72

Statistically significant group differences are highlighted in bold. BMI, body mass index; DASS-21, Depression, Anxiety and Stress Scale – 21 items; ESS, Epworth Sleepiness Scale; ISI, Insomnia Severity Index; MEQ, Morningness-Eveningness Questionnaire; PSQI, Pittsburgh Sleep Quality Index; SE, standard error; TIB, time in bed; TST, total sleep time.

### Sleep

**Figure 2:**
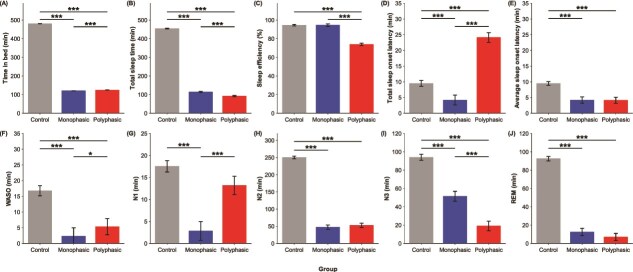
Polysomnographically-assessed sleep. Means and standard errors are depicted for (A) time in bed, (B) total sleep time, (C) sleep efficiency, (D) and (E) sleep onset latency (time taken to reach the first epoch of any sleep stage from lights off), (F) wake after sleep onset (WASO), (G) N1, (H) N2, (I) N3 and (J) REM durations of the control group (gray), the monophasic short sleep group (blue), and the polyphasic short sleep group (red). For the polyphasic short sleep group, (D) the sum and (E) the average of sleep onset latency across all six 20-min sleep opportunities are shown, while for the control and the monophasic short sleep groups, the sleep onset latency of their only sleep period is illustrated. ^*^^*^^*^p < .001, ^*^p < .05 for group contrasts.

Significant group x night interactions were found for all the sleep measures (*p* < .001; Table 2). The significant group x day interaction found for TIB indicates that our sleep opportunity manipulation was successful (*F* = 1647977.10, *p* < .001; Figure 2A). As expected, the control group’s TIB was significantly longer than that of the 2 short sleep groups (*p* < .001). However, there was also a small but statistically significant TIB difference of 3.8 min found between the 2 short sleep groups (*p* < .001), likely a result of the limited interindividual differences in TIB within each group. Despite the slightly longer TIB, TST of the polyphasic short sleep group was 22.3 min shorter than that of the monophasic short sleep group (*p* < .001; Figure 2B), resulting in a significantly lower sleep efficiency (*p* < .001; Figure 2C). The polyphasic short sleep group’s sleep efficiency was also lower than that of the control group (*p* < .001). These group differences in sleep efficiency were largely due to the longer sleep onset latency that the polyphasic short sleep group accumulated over the 6 sleep episodes (*p* < .001; Figure 2D). WASO was also slightly longer in the polyphasic short sleep group as compared to the monophasic short sleep group (*p* = .04; Figure 2F). Notably, when considering sleep onset latency averaged across sleep opportunities, both short sleep groups’ sleep onset latencies were significantly shorter than the control group’s (*p* < .001; Figure 2E), but were not significantly different from each other (*p* = .99).

**Table 2:** Differences in sleep parameters across the 3 groups

	***F***	***P***
TIB	1647977.10	**<.001**
TST	9979.51	**<.001**
Sleep efficiency	31.66	**<.001**
Sleep onset latency (total)	40.91	**<.001**
Sleep onset latency (average)	20.50	**<.001**
WASO	23.54	**<.001**
N1	59.46	**<.001**
N2	672.44	**<.001**
N3	138.10	**<.001**
REM	286.01	**<.001**

Statistically significant group differences are highlighted in bold. REM, rapid eye movement; TIB, time in bed; TST, total sleep time; WASO, wake after sleep onset.

Regarding the sleep stages, the N1, N2, N3, and REM durations of the monophasic short sleep group were significantly shorter than those of the control group (*p* < .001; Figure 2G to J), which is unsurprising given the much shorter TIB. In contrast, while the polyphasic short sleep group had shorter N2, N3, and REM durations than the control group (*p* < .001; Figure 2H–J), these 2 groups did not differ in N1 duration (*p* = .10; Figure 2G).

Comparisons between the 2 short sleep groups showed that the polyphasic short sleep group had significantly longer N1 sleep (*p* < .001; Figure 2G) but shorter N3 sleep (*p* < .001; Figure 2I) as compared to the monophasic short sleep group. This translated to a higher percentage of TST spent in N1 and a lower percentage of TST spent in N3 in the polyphasic short sleep group as compared to the monophasic short sleep group (*p* < .001; Figure S1A and S1C). While N2 duration was similar in both short sleep groups (*p* = .48; Figure 2H), N2 percentage was significantly higher in the polyphasic short sleep group than the monophasic short sleep group (*p* < .001; Figure S1B). REM duration and percentage did not differ between the 2 short sleep groups (*p* = .32 and *p* = .50; Figures 2J and S1D).

**Figure 3:**
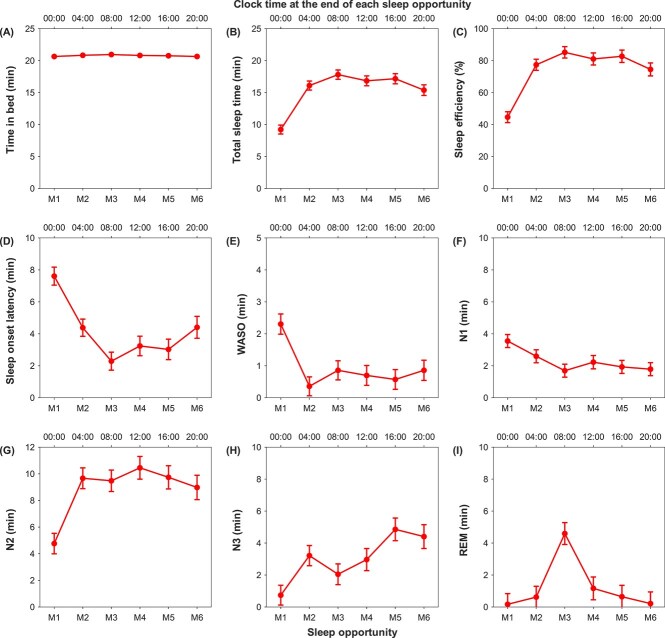
Polysomnographically-assessed sleep in each of the 20-min sleep opportunity of the polyphasic short sleep group. Least square means derived from the general linear mixed model are depicted for (A) time in bed, (B) total sleep time, (C) sleep efficiency, (D) sleep onset latency, (E) wake after sleep onset (WASO), (F) N1, (G) N2, (H) N3 and (I) REM durations across the 6 sleep opportunities (M1–M6). Sleep timings illustrated here are based on a habitual wake time of 08:00. Error bars represent standard errors.

Within the polyphasic short sleep group, significant changes across the 6 sleep opportunities were found for all sleep parameters (*p* < .02; Table 3), except for TIB. That TIB remained stable throughout the manipulation period further demonstrates the success of our sleep manipulation (*F* = 1.75, *p* = .13; Figure 3A). The shortest TST of 9.2 min was observed during the first sleep opportunity (M1), before sharply increasing to 16.1 min during M2 (*p* < .001) and remaining stable for the remaining sleep opportunities (M2 vs M3–6: *p* > .08; Figure 3B). Changes in TST from M1 to M6 are reflected in the changes in sleep efficiency (Figure 3C). Similarly, sleep onset latency and WASO were highest during M1, before dropping during M2 (*p* < .001; Figure 3D and E). Sleep onset latency decreased further during M3 (*p* = .01) before stabilizing during the daytime (M3 vs M4–5: *p* > .20) and increasing slightly in the evening (M3 vs M6: *p* = .01). WASO remained stable from M2 to M6 (*p* > .23).

**Table 3:** Changes in sleep parameters across sleep opportunities in the polyphasic short sleep group

	***F***	***P***
TIB	1.75	.13
TST	19.57	**<.001**
Sleep efficiency	18.40	**<.001**
Sleep onset latency	11.08	**<.001**
WASO	4.89	**<.001**
N1	2.93	**.02**
N2	6.77	**<.001**
N3	5.25	**<.001**
REM	6.36	**<.001**

Significant effects of sleep opportunity are highlighted in bold. REM, rapid eye movement; TIB, time in bed; TST, total sleep time; WASO, wake after sleep onset.

N1 showed a small gradual decrease over the first 3 sleep opportunities, but the difference was only statistically significant at M3 (*p* < .002; Figure 3F). There were no further changes in N1 duration for the remaining sleep opportunities (M3 vs M4–6: *p* > .36). Mirroring the changes in TST, N2 duration increased from 4.8 min during M1 to 9.7 min during M2 (*p* < .001), before remaining unchanged for the rest of the sleep opportunities (M2 vs M3–6: *p* > .48; Figure 3G). N3 duration showed a general increase over the 6 sleep opportunities, from 0.7 min during M1 to 4.4 min on M6 (*p* < .001, Figure 3H). REM duration was minimal for almost all sleep opportunities (0.2–1.2 min), except for M3, where it jumped to 4.6 min (M3 vs M1–6: *p* < .001; Figure 3I).

To summarize, within the polyphasic short sleep group, the first 20-min TIB appeared to be different from the other sleep opportunities, e.g. shortest TST, longest sleep onset latency, and longest WASO (Figure 3). These differences might contribute to some of the differences in sleep parameters between the polyphasic and the monophasic short sleep groups (Figure 2), and may be attributed to the fact that the first 20-min sleep opportunity of the polyphasic group was scheduled slightly before the habitual bedtime of the participants (e.g. 23:40–00:00 for an individual with a habitual 00:00–08:00 sleep schedule), who would, as a result, sleep at a less optimal circadian phase with slightly reduced homeostatic sleep pressure. To better understand the extent to which it may have influenced the overall results, additional group comparisons were conducted without including the data from the first 20-min sleep opportunity of the polyphasic short sleep group on all the sleep parameters, except those involving durations, i.e. TIB, TST, and the duration of each sleep stage, because in these analyses, the reduced total TIB of the polyphasic short sleep group would undoubtedly shorten some of these duration measures. Overall, these additional analyses revealed similar findings. Specifically, as compared to the monophasic short sleep group, the polyphasic short sleep group still had significantly lower sleep efficiency (*p* < .001; Figure S2A), which was driven by the significantly longer sleep onset latency accumulated by the polyphasic short sleep group across the 5 sleep opportunities (*p* < .001; Figure S2B). Average sleep onset latency still did not significantly differ between the 2 short sleep groups (*p* = .50; Figure S2C). We again found that the polyphasic short sleep group had significantly higher N1% and N2% but lower N3% than the monophasic short sleep group (*p* < .001; Figure S2D–F), while the 2 short sleep groups did not differ in REM% (*p* = .67; Figure S2G). In other words, the apparently different properties of the first 20-min sleep opportunity of the polyphasic short sleep group did not have much impact on the findings from our group comparisons.

### Subjective sleepiness

**Figure 4:**
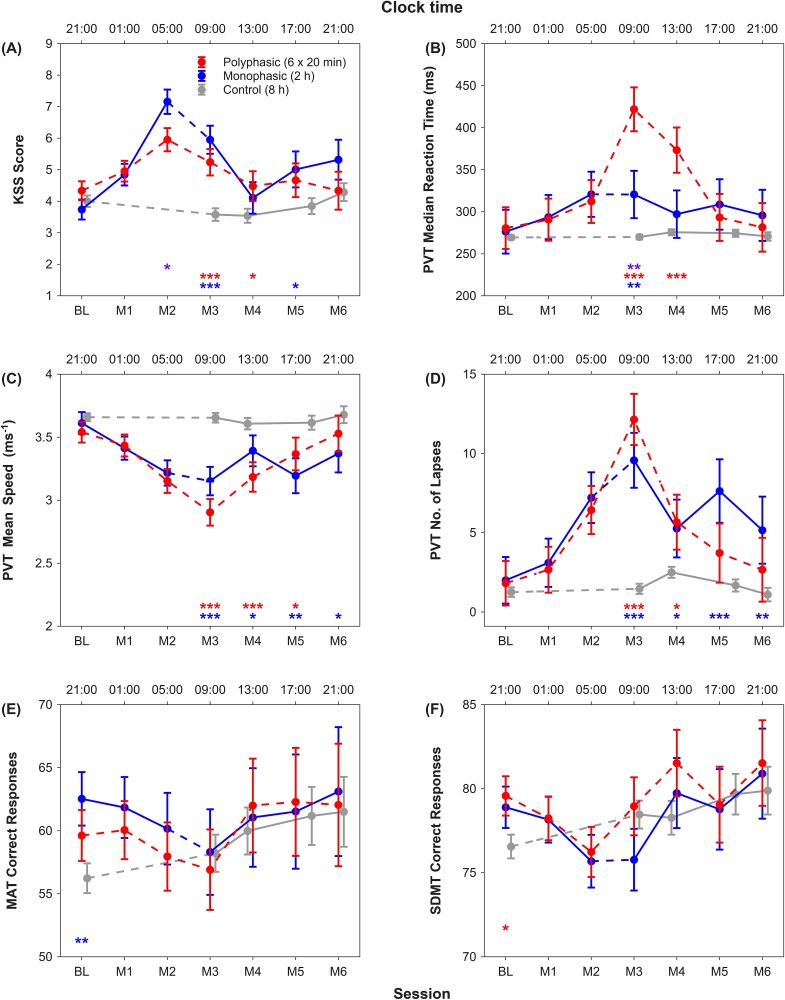

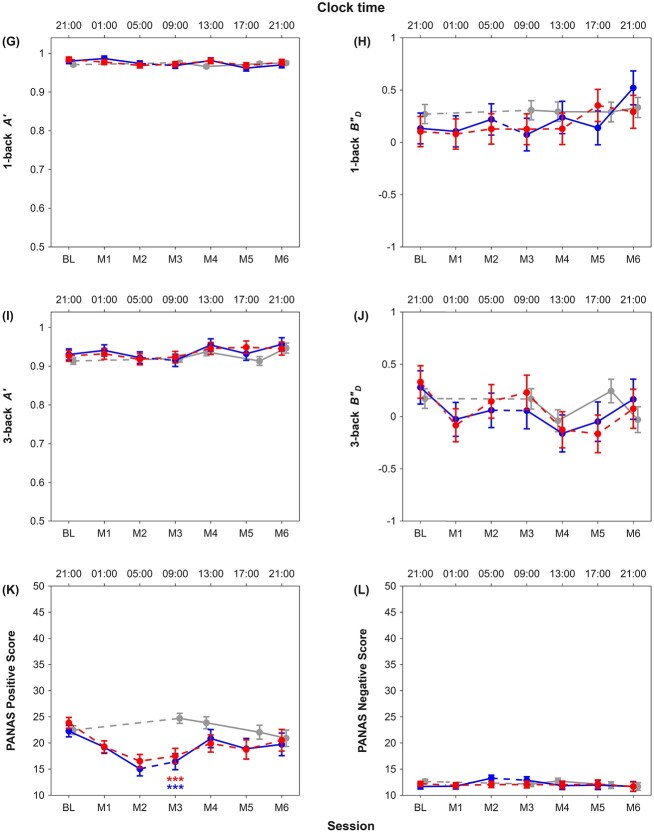
Effects of monophasic and polyphasic short sleep on neurobehavioral functions. Least square means and standard errors were derived with general linear mixed models for the polyphasic short sleep group (red) and the monophasic short sleep group (blue) in (A) subjective sleepiness as assessed by the KSS, vigilance as indicated by (B) median reaction time, (C) mean speed, and (D) the number of lapses in the PVT, processing speed as indicated by the number of correct responses in (E) the MAT and (F) the SDMT, working memory and executive functions as indicated by *A’* and *B”_D_* in the (G and H) 1-back and (I and J) 3-back tasks, as well as (K) positive and (L) negative mood as indicated by the respective subscale scores on the PANAS. Least square means and standard errors of these neurobehavioral outcomes were derived with another general linear mixed model for the control group (gray). ^*^^*^^*^p < .001, ^*^^*^p < .01, ^*^p < .05 for group contrasts from independent-samples *t* tests—purple for polyphasic short vs monophasic short, red for polyphasic short vs control, and blue for monophasic short vs control. Solid lines indicate that participants were awake in between the test sessions, while dotted lines indicate that scheduled sleep periods occurred sometime between the test sessions.

For the 2 short sleep groups, a significant group x session interaction was found for KSS score (*F* = 2.33, *p* = .03; Table 4). Although KSS score peaked early in the morning for both groups, it was significantly higher in the monophasic group than the polyphasic group (*p* = .03; Figure 4A). After the monophasic group had had its 2-h sleep opportunity, KSS dropped prominently in the morning and early afternoon (*p* < .01), when it reached baseline level (*p* = .49), but it was elevated from baseline from late afternoon onwards (*p* < .03). In contrast, the polyphasic short sleep group showed less prominent changes in their KSS score across the study period, and their KSS score was higher than baseline only early in the morning (*p* < .001). As for the control group, the changes in their KSS score across sessions were negligible (*F* = 2.80, *p* = .03; Table 4; Figure 4A).

**Table 4:** General linear mixed models testing neurobehavioral changes across sessions for the 3 groups

	***Monophasic short sleep group vs*** ***polyphasic short sleep group***						***Control group***	
	**Group**		**Session**		**Group × session**		**Session**	
	***F***	***P***	***F***	***P***	***F***	***P***	***F***	***P***
KSS score	0.44	.51	**15.96**	**<.001**	**2.33**	**.03**	**2.80**	**.03**
PVT								
Median RT	1.18	.28	**3.18**	**.005**	1.76	.11	1.41	.23
Mean speed	0.10	.75	**9.27**	**<.001**	1.76	.11	1.10	.36
Number of lapses	0.23	.63	**8.28**	**<.001**	0.95	.46	**3.48**	**.009**
MAT number of correct responses	0.08	.78	1.35	.23	0.16	.99	1.47	.21
SDMT number of correct responses	0.27	.61	**4.03**	**<.001**	0.44	.85	1.96	.10
1-back								
*A’*	0.03	.86	2.03	.06	0.43	.86	1.20	.31
*B”_D_*	0.11	.74	1.07	.38	0.45	.85	0.06	.99
3-back								
*A’*	0.02	.88	1.57	.16	0.26	.96	**2.97**	**.02**
*B”_D_*	0.01	.92	2.01	.07	0.21	.97	1.87	.12
PANAS								
Positive score	0.12	.72	**10.25**	**<.001**	0.35	.91	**2.83**	**.03**
Negative score	0.05	.83	1.08	.37	0.87	.52	0.88	.48

Data from the 3 groups could not be analyzed in the same statistical models because the control group did not do 2 of the test batteries during their scheduled sleep period. Significant effects are highlighted in bold. *A’*, A-prime; *B”_D_*, bias; KSS, the Karolinska Sleepiness Scale; MAT, the Mental Arithmetic Test; PANAS, the Positive and Negative Affect Schedule; PVT, the Psychomotor Vigilance Task; RT, reaction time; SDMT, the Symbol Digit Modalities Test.

When comparing the subjective sleepiness levels of the 2 short sleep groups against that of the control group (Figure 4A), we found no significant difference in KSS score at baseline (*p* > .34). In the morning after both the monophasic short sleep group and the control group had respectively had their 2-h and 8-h sleep opportunity, KSS score was clearly higher in the monophasic short sleep group (*p* < .001). At that time of day, after having 3 of their scheduled 20-min sleep periods, KSS score of the polyphasic short sleep group was also higher than that of the control group (*p* < .001). The 2 short sleep groups also had higher sleepiness levels than the control group later in the day (M4 for the polyphasic short sleep group: *p* < .05; M5 for the monophasic short sleep group: *p* = .03).

### Vigilance

Although the group x session interaction for median reaction time in the PVT did not reach statistical significance (*F* = 1.76, *p* = .11; Table 4), the polyphasic short sleep group was considerably slower in their responses in the morning than the monophasic short sleep participants who just had their sleep opportunity (*p* = .009; Figure 4B). In fact, the monophasic short sleep group’s median reaction time remained at the baseline level throughout the study period (*p* < .23). In contrast, the polyphasic short sleep group’s median reaction time remained at baseline level during late night and early morning hours (*p* > .37), but showed a sharp increase in the morning (M2 vs M3: *p* = .002), and remained elevated from baseline in the early afternoon (*p* = .01). It returned to baseline level from late afternoon onwards (*p* > .73). Regarding the other 2 PVT indices – mean speed and the number of lapses, no statistically significant group x session interaction was found (*F* < 1.76, *p* < .11; Table 4). The significant main effects of session (*F* > 8.28, *p* < .001; Table 4) indicated that for both groups, mean speed was the slowest and the number of lapses was the greatest in the morning, improved from morning to early afternoon (*p* < .05), and thereafter, remained stable (*p* > .11; Figure 4C and D). For the control group, vigilance performance was largely stable across sessions (median reaction time: *F* = 1.41, *p* = .23; mean speed: *F* = 1.10, *p* = .36; Table 4), except for the number of lapses (*F* = 3.48, *p* = .009; Table 4), which slightly, but significantly, increased from the morning to early afternoon (*p* = .02) and then returned to baseline level late afternoon (*p* = .36).

The 2 short sleep groups showed similar PVT performance as the control group at baseline (*p* > .10; Figure 4B to D). The 3 indices uniformly revealed that both monophasic and polyphasic short sleep schedules impaired vigilance in the morning (*p* < .001). For the remaining time points, the polyphasic short sleep group showed poorer vigilance than the control group until late afternoon (*p* < .05), while for the monophasic short sleep group, both mean speed and number of lapses revealed poorer vigilance than the control until the end of the protocol (*p* < .05), but no significant difference against the control group was found for median reaction time from early afternoon onwards (*p* > .07).

### Processing speed

Processing speed, as evaluated with the SDMT and the MAT, did not show any significant difference between the 2 short sleep groups (main effect of group: *F* < .27, *p* > .61), and the group x session interactions were also not statistically significant (*F* < .44, *p* > .85; Table 4). The main effect of session was not statistically significant for MAT (*F* = 1.35, *p* = .23; Figure 4E), but was significant for SDMT (*F* = 4.03, *p* < .001), indicating that both the monophasic and the polyphasic short sleep groups had the lowest number of correct responses early in the morning (Figure 4F). Over its sleep episode, the monophasic short sleep group did not show any improvement in SDMT performance (*p* = .96), and improvement was delayed until early afternoon (*p* = .009). Similarly, the polyphasic short sleep group’s SDMT performance did not change in the morning (*p* = .09) and only significantly improved in the afternoon (*p* = .002). For the rest of the wake period, SDMT performance of both short sleep groups was stable (*p* > .13). As for the control group, the improvement in performance across sessions did not reach statistical significance for either MAT (*F* = 1.47, *p* = .21) or SDMT (*F* = 1.96, *p* = .10). At baseline, compared to the control group, the monophasic short sleep group and the polyphasic short sleep group had slightly better MAT (*p* = .009) and SDMT performance (*p* = .03) respectively. No other significant contrast against the control was found at other time points (*p* > .11).

### Working memory and executive functions

Considering the performance of the 2 short sleep groups, none of the main effects or the group × day interactions was statistically significant for *A’* or *B”_D_* in either the 1- or the 3-back tasks (*F* < 2.03, *p* > .06; Table 4; Figure 4G–J). The control group also did not show any significant change in n-back performance (*F* < 1.87, *p* > .12; Table 4), except for *A’* in 3-back (*F* = 2.97, *p* = .02; Figure 4I) which showed subtle, but statistically significant, differences across sessions.

Performances of both the monophasic and the polyphasic short sleep groups were similar to that of the control group at all time points in both 1- and 3-back (*p* > .05). Of note, *A’* in 1-back was close to perfect in all 3 groups, pointing to ceiling effects.

### Mood

Regarding positive mood of the 2 short sleep groups, the group × session interaction effect was not statistically significant (*F* = .35, *p* = .91; Table 4). Together with the significant main effect of session (*F* = 10.25, *p* < .001; Table 4), our data showed similar changes in positive mood in both short sleep groups during the study. Relative to baseline, positive mood of both short sleep groups decreased to the lowest level early in the morning (*p* < .05), remained at this level in the morning (*p* > .37), and improved early afternoon (*p* < .02). For the control group, positive mood also changed significantly across sessions albeit to a much smaller extent (*F* = 2.83, *p* = .03; Table 4; Figure 4K).

Positive mood of the short sleep groups was similar to that of the control group at baseline (*p* > .35). Both the monophasic and the polyphasic short sleep groups had lower positive mood than control in the morning (*p* < .001), but these differences were no longer observed later in the day (*p* > .06).

For the 2 short sleep groups, no significant main effects of group and session, as well as their interaction, were found for negative mood (*F* < 1.08, *p* > .37; Table 4). Negative mood was also stable across sessions for the control group (*F* = .88, *p* = .48; Table 4). The negative mood scores of the 2 short sleep groups did not significantly differ from those of the control group at all times of day (*p* > .21; Figure 4L). In fact, negative mood scores remained at a low level throughout the study period for all 3 groups.

## Discussion

The present study showed that in young adults, adopting the “Uberman” sleep schedule over a 24-h period reduced subjective alertness, vigilance, and positive mood relative to a well-rested state, in stark contrast to the optimized performance and mood proposed by polyphasic sleep advocates [9] and what many aspire to achieve when they sleep little. In fact, the impairing effect of this polyphasic short sleep schedule on vigilance, particularly in the morning, was beyond what a monophasic short sleep schedule of the same total TIB would induce and hence, greater than the effect of curtailed sleep alone. Furthermore, the poorer neurobehavioral functions of the monophasic and the polyphasic short sleep groups, together with the stable and optimal state achieved by the control group, highlight the importance of obtaining the age-appropriate sleep duration for effective functioning throughout the waking period.

The neurobehavioral deficits shown by both short sleep groups might be attributable to their prominent sleep-related differences from the control group. Both short sleep groups, by design, had much shorter TSTs than the control group, and thus experienced greater sleep pressure, as evidenced by the shorter sleep onset latency during each sleep episode. WASO was also shorter in the short sleep groups as compared to the control group. Notably, relative to the monophasic short sleep schedule, the polyphasic short sleep schedule resulted in even more curtailed sleep, poorer sleep efficiency, longer total awakenings both at the beginning of the sleep opportunities (i.e. sleep onset latency) and after sleep onset (i.e. WASO), and greater proportion of lighter sleep stages (N1% and N2%), but reduced N3%.

### Neurobehavioral effects of monophasic short sleep

The control participants in our study were well-rested. Although their PSG data were from the first night of our laboratory-based study, first night effect, if any, appeared to be minimal, since out of the 8-h TIB, their average TST was 454.3 min, and sleep efficiency was 94.5%; moreover, their mean sleep latency was 9.5 min, WASO was 16.8 min, and the percentages of N1, N2, N3, and REM sleep were 3.9%, 55.1%, 20.1%, and 20.4%. In addition, their KSS score, PVT performance, and 1- and 3-back performance were similar to the levels achieved by participants having multiple nights of 9- to 10-h TIB in previous studies [42, 47]. Their largely stable performance across the 16-h post-sleep waking period was consistent with the reduced circadian modulation of neurobehavioral functions when individuals are not sleep-deprived (i.e. when homeostatic sleep pressure is low) [48].

In contrast, when homeostatic sleep pressure was high [48], as evidenced by the shorter sleep onset latency and WASO observed in our monophasic short sleep group relative to the control group, neurobehavioral changes across sessions were more prominent. As this group had stayed up for 21 h until session M2 early in the morning, the high sleep pressure, together with the increased circadian drive for sleep [49], elevated subjective sleepiness, degraded vigilance as well as speed of processing, and reduced positive mood. After their 2-h nocturnal sleep opportunity, subjective alertness and other neurobehavioral functions of the monophasic short sleep group differed in their trajectories. Vigilance reached the lowest level after these participants woke up in the morning, indicating that following its short sleep opportunity, this group’s homeostatic sleep pressure might not have fully dissipated and could not be offset by the increasing circadian drive for wakefulness at that time of day [49]. For the same reasons, speed of processing and positive mood did not improve across the sleep period. In contrast to these neurobehavioral measures, subjective alertness improved over the 2-h sleep episode. The dissociation between objective measures of cognitive performance and subjective alertness has been uncovered in multi-night sleep restriction studies [26, 50]; therefore, our findings reinforce the idea that sleep-restricted individuals may not be able to reliably estimate the extent to which their objective cognitive functions are impaired, and point out that this seemed to be particularly the case in the morning after a night of sleep curtailment. In the early afternoon, probably due to further increases in the circadian wake-promoting signals, improvements in subjective alertness, vigilance, speed of processing, and positive mood were observed. Dissociation in the neurobehavioral measures emerged again in the late afternoon and evening, when cognitive performance and positive mood were stable, but subjective sleepiness increased. This might indicate that subjective alertness was particularly sensitive to the increased homeostatic sleep pressure during the day, especially when it might not have dissipated effectively the night before, despite the increasing circadian wake-promoting signals during the biological day [49].

Compared to the levels achieved after a night of 8-h TIB, following a night of 2-h TIB, subjective alertness was lower in the morning and late afternoon, positive mood was poorer in the morning, and depending on the measures considered, vigilance could be impaired throughout the wake period. It is noteworthy that a previous study reported increased subjective sleepiness and reduced vigilance only after having a 3-h TIB for 2 nights [47]; with TIB further shortened to 2 h in our study, we found that 1 night was sufficient to degrade subjective alertness and vigilance. In fact, a recent meta-analysis has revealed that subjective alertness and vigilance were the 2 neurobehavioral domains that were impaired after 1 night of partial sleep deprivation, while working memory, inhibitory control—an element of executive functions, and speed of processing were not affected [51]. Although we did not find any difference in performance in the speed of processing and working memory / executive function tasks between the monophasic short sleep group and the control group during the daytime, with more nights of sleep curtailment, the cumulative impairment on these functions will likely become more noticeable [26, 37, 50].

Negative mood remained at a low level throughout the study period for both the monophasic short sleep group and the control group. Together with similar observations from previous sleep restriction studies [26, 37–39, 42], our finding highlighted that this neurobehavioral domain, at least when assessed with the PANAS, might not be sensitive to the effects of partial sleep deprivation. As shown in a recent meta-analysis, among all the emotions studied, positive mood had the largest relationship with sleep duration, while the association between negative mood and sleep duration was smaller [52].

### Neurobehavioral effects of polyphasic short sleep

Our data clearly showed that polyphasic short sleep did not optimize and maintain neurobehavioral functions as advocated. From the evening to the early morning, i.e. a period overlapping mostly with the biological night when one typically sleeps in an entrained condition, despite the two 20-min sleep opportunities afforded by the “Uberman" sleep schedule, degradation in subjective alertness, vigilance, speed of processing, and positive mood were observed, suggesting that the 2 short TIBs, which consisted of a total of 4.0 min of N3 (Figure 3H), were not sufficient to completely dissipate homeostatic sleep pressure to counteract the increasing circadian drive for sleep. In the morning, increases in the circadian wake-promoting signals might have ameliorated the impact of high sleep pressure, and as a result, subjective alertness, speed of processing, and positive mood did not deteriorate further; however, further impairment in vigilance was observed as all 3 PVT indices reached their worst levels during the morning hours. This might imply that vigilance was particularly sensitive to the build-up of homeostatic sleep pressure during the “Uberman” sleep schedule and could only be offset by a stronger circadian drive for wakefulness later in the day. Indeed, with the increasing circadian wake-promoting signals during the daytime, vigilance and speed of processing improved in the afternoon and remained at baseline level for the rest of the waking period, when subjective alertness and positive mood were also found to be at well-rested levels.

Another evidence that the sleep episodes of the “Uberman” sleep schedule were not sufficient to dissipate sleep pressure and optimize neurobehavioral functions is that late at night and early in the morning (i.e. in sessions M1 and M2), the 2 short sleep groups did not show any difference in cognitive performance or positive mood, although the polyphasic short sleep group had already had 1–2 of its short sleep opportunities, while the monophasic short sleep group had stayed awake for 17–21 h and should have accumulated greater homeostatic sleep pressure. Therefore, while the total duration of 4.0 min of N3 from the first 2 episodes of the polyphasic short sleep group appeared to be able to lead the polyphasic short sleep group to feel less sleepy than the monophasic short sleep group, these 20-min sleep episodes were not sufficient to reduce the deficits in cognitive performance caused by extended wakefulness, and the total duration of 0.8 min of REM sleep did not appear to benefit mood. This again highlighted the dissociation between subjective alertness levels and cognitive performance in sleep-restricted individuals [26, 50].

Our results appeared to be in contrast to those by Rosenblum et al.’s [23] who found limited impact on a range of cognitive functions, including vigilance, when they compared the performance of their participant who followed the “Uberman” sleep schedule for 5 weeks against an 8-h TIB monophasic control group. However, it is noteworthy that relative to baseline, their polyphasic participant showed a 1.75-fold increase in the number of PVT lapses. While it was not clear at what time of day this task was administered in Rosenblum et al.’s study, our data suggest this somewhat attenuated deficit in vigilance under the “Uberman” schedule could be observed early in the biological night or in the evening (1.47-fold increase from the baseline PVT lapse value at 1.81; Figure 4D); on the other hand, if vigilance was assessed at other times of day, deficits could be more prominent (at least 2.05-fold increase from baseline).

The deleterious impact of polyphasic short sleep on neurobehavioral functions appeared to be beyond the effects of sleep deficiency alone. As shown by the poorer vigilance of the polyphasic than the monophasic short sleep group in the morning, splitting up the already severely curtailed sleep opportunity into multiple short sleep episodes could lead to even greater neurobehavioral deficits than those induced solely by sleep curtailment. This was particularly so in the early part of the day. In contrast, in the late afternoon and evening, when the monophasic short sleep group had stayed awake for more than 9 h and showed elevated sleepiness, the 20-min sleep bouts in the “Uberman” sleep schedule, which yielded a total of 32.5 min of TST, were able to keep subjective alertness at baseline levels. This is in line with our previous work, which revealed that splitting up a less curtailed sleep opportunity of 6.5 h into 2 sleep periods—a core sleep period of 5 h and a 1.5-h daytime nap—was able to reduce sleep loss-induced deficits in subjective alertness because the nap opportunity helped reduce homeostatic sleep pressure [39]. However, it is important to note that in that previous study, this benefit of split sleep was also found for vigilance, working memory/executive functions, speed of processing, and positive mood. This implies that among sleep-restricted individuals, longer and more consolidated sleep during the daytime might result in more prominent benefits on neurobehavioral functions in the latter half of the day.

In the current study, it could be argued that the more prominent deficit in morning vigilance of the polyphasic short sleep group, relative to the monophasic short sleep group, was due to the shorter TIBs of the polyphasic group (3 × 20-min TIB vs 2-h TIB) by the morning test session. It is possible that if the 2-h TIB of the monophasic short sleep group were to be shifted from the middle to the end of the manipulation period, the monophasic short sleep group could have accumulated a much higher sleep pressure and more degradation in neurobehavioral functions, which could help showcase the benefits of the “Uberman” sleep schedule in attenuating sleep pressure and minimizing sleep loss-induced neurobehavioral deficits, as shown in previous studies that compared performance during a night of total sleep deprivation with and without regular short sleep episodes (e.g. 80 min sleep after each 160-min wake period) [11, 12]. Along this line, the lower subjective sleepiness level of the polyphasic vs the monophasic short sleep group early in the morning could be due to the two 20-min sleep opportunities of the former and the sustained prior waking duration of the latter. That said, later in the day, when the monophasic short sleep group showed reduction in subjective alertness, the short sleep episodes of the polyphasic group appeared to be able to keep subjective alertness level at baseline levels. To rule out the possibility that neurobehavioral differences between the polyphasic short sleep schedule and the monophasic short sleep group were due to differences in prior total TIBs, or in other words, sleep history, future studies can involve multiple monophasic short sleep groups such that in each neurobehavioral test session, performance of the polyphasic short sleep group can be compared with a corresponding monophasic group that had had the same total prior TIBs, e.g. 2 episodes of 20-min TIB vs one episode of 40-min TIB in session M2, 3 episodes of 20-min TIB vs one episode of 60-min TIB in session M3, and so on.

Of note, the limited differences in speed of processing, working memory/executive functions, negative mood between the polyphasic short sleep group and the control group in our study should not be interpreted as a benefit of the “Uberman” sleep schedule. As discussed in the section about monophasic short sleep, the impairing effects on some daytime functions might not manifest until after more nights of sleep curtailment. Furthermore, negative mood, as measured by PANAS, was not affected in prior studies involving different extents of sleep curtailment for multiple nights [26, 37–39, 42], and here, we extended the null finding to the “Uberman” sleep schedule.

Overall, the degraded neurobehavioral functions observed in the polyphasic short sleep group during the biological night suggest that at night, when circadian drive for sleep is high, it is better to sleep throughout this period rather than to stay awake with intermittent short sleep bouts and engage in cognitive activities at reduced capability. Even during the daytime, despite the multiple short sleep episodes in the “Uberman” sleep schedule, alertness, vigilance, and positive mood were poorer relative to the well-rested state demonstrated by the control group. Hence, there is little doubt that most individuals will face challenges to maintain their cognitive performance and psychological well-being at least during the initial period when they adopt this polyphasic short sleep schedule. This is further supported by Rosenblum et al.’s study, where even among individuals who were initially highly motivated to follow the “Uberman” sleep schedule, none completed the intended 8-week period because of its undesirable influence on their professional or private life, and cognitive functions [23]. Furthermore, an association between polyphasic sleep and higher levels of daytime sleepiness has been reported in an observational study [10].

### Sleep architecture effects of polyphasic short sleep

As the only study to have compared the sleep architecture associated with the “Uberman” sleep schedule with those in a well-rested schedule and a monophasic short sleep schedule of the same total TIB, this study found clear alterations in sleep architecture when individuals split their already shortened TIB into multiple sleep opportunities scattered across a 24-h period.

Firstly, as compared to a night of 8-h TIB, because of its substantially reduced total TIB, the “Uberman” sleep schedule resulted in remarkable reductions in TST and the duration of each sleep stage, except N1. The total N3 duration and REM duration over a 24-h period were merely 18.2 min and 7.4 min, respectively, which are too short to be justified as the “scientifically required amounts” of N3 and REM sleep proposed by the polyphasic sleep advocates. For example, a previous study where participants were selectively deprived of SWS over 2 nights, even when their SWS duration was reduced to only 44.9–61.9 min, notable increases in objective sleepiness were observed [53]. Like Rosenblum et al. [23], we found that relative to an 8-h TIB, the “Uberman” schedule resulted in a lower proportion of REM (Figure S1D: mean difference = -13.3%; -7% in reference [23]), but no change in N2% (Figure S1B: mean difference = 2.5%; 1% [23]); however, while Rosenblum et al. reported no change in N1% (1%) and increased N3% (4%) during the “Uberman” schedule, our polyphasic short sleep group had greater N1% (Figure S1A: mean difference = 10.5%) but similar N3% (Figure S1C: mean difference = 0.3%) as the control group. These differences between studies could be due to the different sample sizes (*n* = 1 vs 73), as well as the participants’ sleep history: while our participants were well rested before being exposed to the “Uberman” schedule, the participant in Rosenblum et al.’s study had been practicing polyphasic sleep for 5 weeks, and likely was carrying greater homeostatic sleep pressure. In our study, other than differences in sleep stages, sleep onset latency in each sleep opportunity and total WASO were reduced in the polyphasic short sleep group as compared to the control group, probably because the polyphasic short sleep group had accumulated greater homeostatic sleep pressure over the longer scheduled wake durations.

These sleep-related alterations in the “Uberman” schedule were not simply due to sleep curtailment. Comparisons against the monophasic short sleep group with the same total TIB have revealed that splitting a short sleep opportunity into multiple episodes across the 24-h day resulted in less TST mainly due to the increased time spent falling asleep: although each of the six 20-min sleep opportunities was associated with relatively short sleep onset latencies (maximum duration of 7.6 min), participants with the polyphasic sleep schedule had to go to sleep 6 separate times, rather than just once, which caused sleep onset latency to accumulate to 24.9 min, a huge increase compared to the 4.2 min of the monophasic short sleep group. Since WASO was also longer in the polyphasic than the monophasic short sleep group, the “Uberman” schedule resulted in a much lower sleep efficiency, which was, in fact, 73.9% on average. Of note, except for the 20-min sleep opportunity in the morning, sleep efficiency of all the other sleep episodes was below 85%, indicating poor sleep quality. Moreover, individuals adopting the “Uberman” schedule spent a greater proportion of their TST in lighter sleep stages but less time in N3 than the monophasic short sleep group. A possible reason for this is that the short length of each sleep opportunity was not always sufficient to allow for the transition to N3. However, it should be noted that N3 sleep duration increased over the 6 sleep opportunities, likely as a response to the increasing homeostatic sleep pressure. This suggests that if the Uberman schedule is followed for a longer period of time, N3 sleep may be further prioritized and make up a larger percentage of TST. As for REM sleep, the 2 short sleep schedules did not show any significant difference in total duration or proportion. REM sleep duration remained low for most of the 20-min sleep opportunities, and the surge in REM sleep duration in the morning was likely a result of the stronger circadian REM drive that happens in the late biological night/early biological morning [54, 55].

### Limitations and future studies

Our study had a few limitations. Firstly, the monophasic and the polyphasic sleep schedules were only implemented for a 24-h period. Important research questions remain to be addressed in future studies, including whether neurobehavioral deficits will accumulate in individuals adopting polyphasic sleep schedules for prolonged periods, whether interindividual differences exist in the adaptation to these sleep schedules, as well as the factors underlying these interindividual differences. Secondly, the 2-h TIB of the monophasic short sleep group ended at the participants’ habitual wake time. As mentioned previously, if the sleep opportunity was placed at other time points, the contrasts with the polyphasic short sleep group might yield different conclusions; this also holds true for varying the timings of the short sleep episodes under the polyphasic schedule. Similarly, we only studied the “Uberman” sleep schedule, and did not investigate polyphasic sleep schedules that involved a core nocturnal sleep period and shorter daytime naps, e.g. the “Everyman” sleep schedule, which comprises a 3-h nocturnal sleep opportunity and three 20-min daytime sleep episodes. More work is required to better characterize the neurobehavioral benefits, if any, associated with various polyphasic short sleep schedules. Thirdly, our sample consisted of young adults. Since neurobehavioral responses to both sleep restriction [56], as well as split sleep without sleep deficiency (sleep:wake ratio = 1:2) [57] differ across age groups, future studies should explore the possibility that polyphasic short sleep may differentially affect young and older individuals. Fourthly, we only studied basic neurobehavioral functions. The impact of polyphasic short sleep on other cognitive domains, e.g. decision making, is yet to be investigated.

## Conclusions

Many individuals struggle to have sufficient sleep and hope to minimize their time-in-bed in order to maximize their waking periods for study and work. Multiple polyphasic sleep schedules, including the “Uberman” schedule, have been proposed to enable prolonged wakefulness while retaining a “scientifically required amount” of SWS and REM sleep, and thus, can optimize performance and mood. Our study showed that the “Uberman” schedule substantially shortened total sleep duration, N2, N3, and REM sleep, not just simply as a consequence of curtailed TIB, but also because under this schedule, sleep had to be initiated multiple times across the 24-h period, thereby drastically increasing the cumulative sleep onset latency by 20 min in total. This “cost” for multiple sleep onset was high, given that the total TIB was only 2 h per 24 h. These sleep changes might explain why this polyphasic sleep schedule might induce greater neurobehavioral deficits, especially in vigilance in the morning, than a monophasic sleep schedule of the same total TIB. Overall, individuals will likely struggle cognitively and emotionally during both day and night, at least when they first start adopting this sleep schedule, similar to what is proposed by many proponents of the polyphasic sleep schedule. However, lengthier protocols are required to determine the validity of the proposal that these suboptimal effects will normalize after this initial period, and characterize the long-term health consequences of polyphasic short sleep, other than suppressed growth hormone release reported by Rosenblum et al. [23]. On the other hand, the stable daytime functioning of the control group with an 8-h nocturnal TIB highlights that optimal neurobehavioral functions that are desired by many can be achieved by having an age-appropriate amount of sleep.

## Supplementary Material

Supplementary_Materials_20260202_zsag031

## Data Availability

The data underlying this article will be shared on reasonable request to the corresponding author.
