# Supplementary material for: Neurobehavioral functions and sleep architecture during polyphasic and monophasic short sleep schedules
Source: Sleep. 2026 Feb 5;49(5):zsag031. doi: 10.1093/sleep/zsag031 (PMC13163185; doi:10.1093/sleep/zsag031)

**Supplementary Material**

**Neurobehavioural functions and sleep architecture during polyphasic and monophasic short sleep schedules**

Tiffany B. Koa^1,2^; June C. Lo^1,2,3^

^1^Centre for Sleep and Cognition, Yong Loo Lin School of Medicine, National University of Singapore, Singapore

^2^Human Potential Translational Research Programme, Yong Loo Lin School of Medicine, National University of Singapore, Singapore

^3^Department of Medicine, Yong Loo Lin School of Medicine, National University of Singapore, Singapore

Corresponding author:

Dr. June Chi-Yan Lo

Centre for Sleep and Cognition

Yong Loo Lin School of Medicine,

National University of Singapore,

MD1 Tahir Foundation Building, 12 Science Drive 2,

Singapore 117549

Phone: (+65) 66016146

E-mail: june.lo@nus.edu.sg

Figure S1. Percentage of sleep stages as assessed with polysomnography. Means and standard errors are depicted for **(A)** N1%, **(B)** N2%, **(C)** N3%, and **(D)** REM% of the three groups. *** *p* < .001, ** *p* < .01, * *p* < .05 for group contrasts.


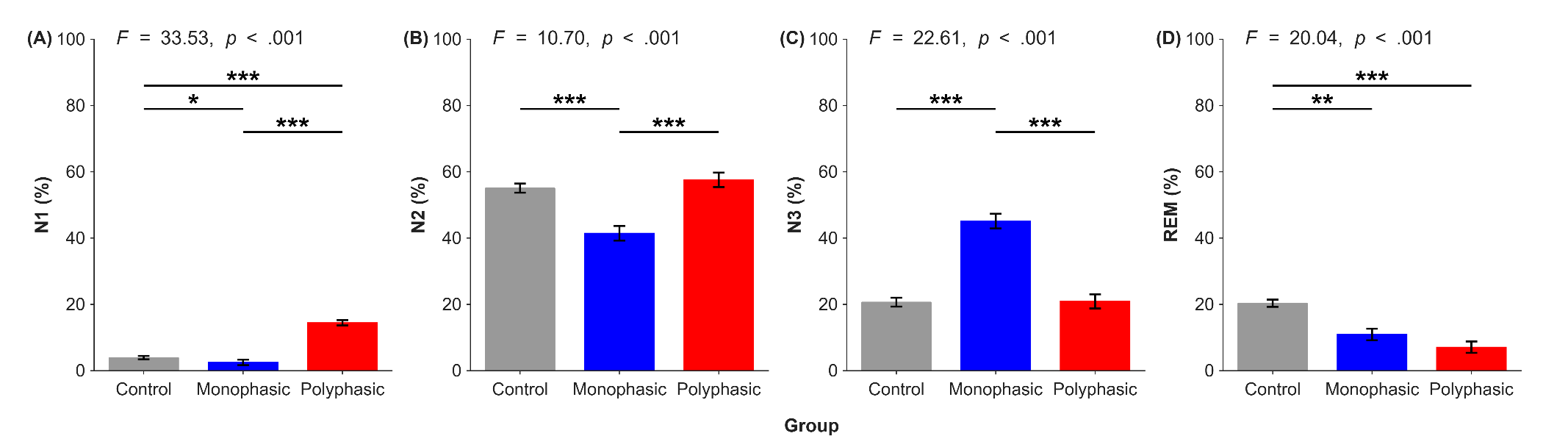


Figure S2. Polysomnographically-assessed sleep. Data for the polyphasic short sleep group excludes those from the first 20-min sleep opportunity. Means and standard errors are depicted for **(A)** sleep efficiency, **(B)** total sleep onset latency, **(C)** average sleep onset latency, **(D)** N1%, **(E)** N2%, **(F)** N3%, and **(G)** REM%, of the three groups. *** *p* < .001, ** *p* < .01, * *p* < .05 for group contrasts.


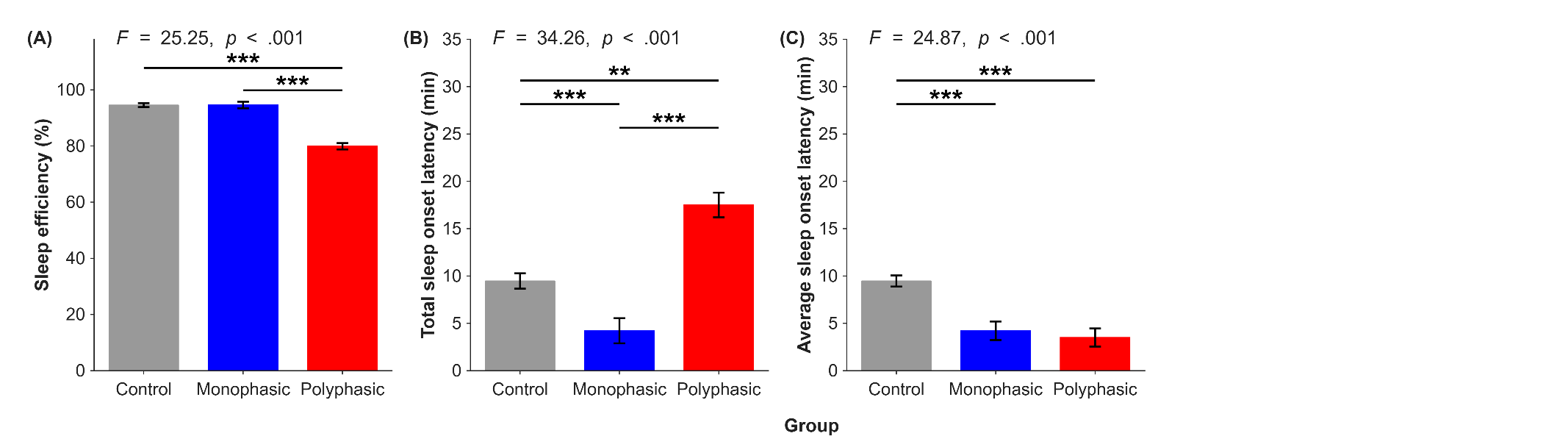

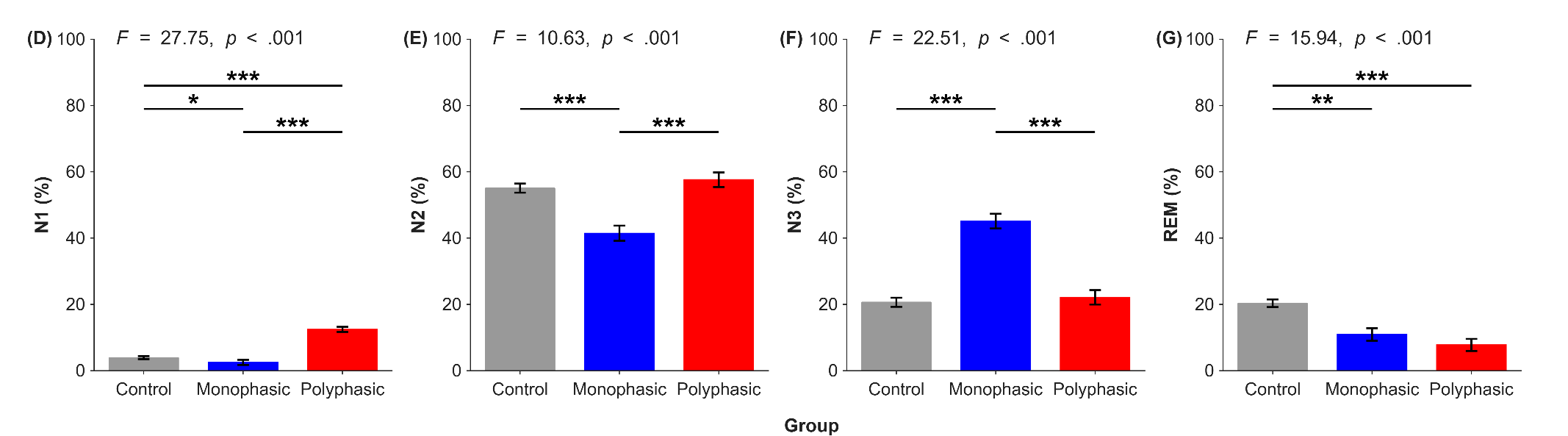

Supplement: Supplementary_Materials_20260202_zsag031 [file supplementary_materials_20260202_zsag031.docx]
